# Perceived Orthodontic Needs and Attitudes towards Early Evaluation and Interventions: A Survey-Based Study among Parents of Italian School-Aged Children

**DOI:** 10.3390/clinpract14030092

**Published:** 2024-06-18

**Authors:** Federica Di Spirito, Davide Cannatà, Valentina Schettino, Marzio Galdi, Rosaria Bucci, Stefano Martina

**Affiliations:** 1 Department of Medicine, Surgery and Dentistry “Scuola Medica Salernitana”, University of Salerno, Via Allende, 84081 Baronissi, SA, Italy; fdispirito@unisa.it (F.D.S.); v.schettino14@studenti.unisa.it (V.S.); marzio.galdi@gmail.com (M.G.); 2 Department of Neurosciences, Reproductive Sciences and Oral Sciences, University of Naples Federico II, Via Pansini 5, 80131 Naples, NA, Italy; rosaria.bucci@unina.it

**Keywords:** children, dental practice, early diagnosis, early orthodontic treatment, interceptive orthodontics, pediatrics

## Abstract

(1) Background: Parents’ awareness of malocclusion and their attitude towards early evaluation and interventions influence children’s orthodontic management. This cross-sectional study investigates factors that affect the perceived orthodontic needs and the attitude towards interceptive orthodontics among a sample of one-thousand eight-hundred and six (1806) parents of children aged between 6 and 11 years. (2) Methods: The investigation was carried out thought a 18-items online questionnaire divided as follows: characteristics of respondents; perceived child’s orthodontic needs; attitude toward early orthodontic evaluation and interventions. The associations between responses were assessed with a Chi-square test. (3) Results: Two-thirds of the respondents referred to having consulted an orthodontist for their child, and more than half of them initiated the required orthodontic treatment. In 44% of cases, the orthodontic consultation occurred after the age of 7 years. Parents’ higher education and history of orthodontic treatment were associated with a greater awareness of orthodontic needs. Parents’ perception of the impact of teeth on their child’s personality was significantly associated with the decision to start the orthodontic treatment (*p* < 0.001). (4) Conclusions: Although the parents’ level of awareness of their child’s orthodontic needs was generally satisfactory, the results of the present study pointed out the need for a better education regarding the importance of an early orthodontic assessment.

## 1. Introduction

Orthodontic needs, namely malocclusions or associated risk factors, affect more than a half of the children and adolescents in the world, and thus they represent a relevant health problem, as well as an economic burden for either health public services or families caring for young children [1].

This high prevalence was also commonly observed in the Italian pediatric population [2,3,4,5,6]. Notably, less than 20% of children aged between 2 and 7 years, and about 10% between the age of 8 and 13 years, are free from issues in tooth alignment, facial skeletal development or oral functions [7].

While malocclusions observed in children with deciduous or early mixed dentition were mostly of mild severity, three out of four subjects with late mixed or permanent dentition had moderate or severe orthodontic issues [7]. Indeed, while mild developing malocclusions can remain unchanged over time when risk factors are corrected, some of them can worsen with growth, turning into more severe dentofacial deformities [8].

Since dentofacial features play an essential role in social integration, such deformities have been recognized to be a major health problem, impairing personal interactions, and sometimes resulting in the occurrence of psychological disorders [9]. Conversely, an aesthetic occlusion results in a more attractive appearance, promoting increased self-esteem and acceptance by peers [10]. The above also applies to children, since those with dentofacial deformities are stated to be subjected to teasing, bullying, and social rejection [11].

The early elimination of any primary etiological component of malocclusions, such as oral bad habits, loss of space in the dental arch due to caries, teeth crowding, anomalies in the eruption patterns, transverse and sagittal skeletal discrepancies, could prevent the worsening of developing deformities [12]. Regarding this, favorable results with good long-term stability can be obtained with interceptive orthodontic approaches, aiming at removing the risk factors of malocclusion, promoting favorable dentofacial growth and possibly preventing unfavorable one [13]. As a result, such interventions potentially streamline or erase the complexity of more extensive therapy in the future, minimizing overall treatment time and cost [14].

Dentoskeletal and functional issues requiring a timely management can be easily detected by a specialist, who can assess the need for interventions, and thus timely referring patients to an orthodontic evaluation appears to be crucial to intercept malocclusion as soon as possible [7]. Consistently, the American Association of Orthodontists (AAO) recommends starting orthodontic check-ups ideally at the age of 7 years, i.e., when the transition period from deciduous to permanent dentition is about to start or has just begun [3].

As parents are a key gatekeeper to treatment access [15], parental awareness of the importance of time-appropriate orthodontic evaluation and interventions plays a crucial role in children’s orthodontic management. When parents have accurate and comprehensive information about early orthodontic interventions, they can timely refer their children to the orthodontist, directly contributing to facilitating the management of any issues. Conversely, inadequacy in parental information and awareness may lead to failure in quickly pursuing the orthodontic therapies required for the child [16].

Previous studies conducted in different countries of the world identified perceived dental and orthodontic needs, i.e., problems in the alignment of teeth, skeletal and functional issues, as the main factor affecting parents’ attitude towards seeking early orthodontic interventions for their children [17,18,19,20].

Meanwhile, the perception of orthodontic problems proved to be positively associated with parents’ level of literacy and parental history of orthodontic treatment [16,18,21,22].

To the best of our knowledge, limited data exist regarding factors that affect the perception of children’s orthodontic needs, and the parental propensity to seek an early orthodontic intervention in Italy. Therefore, the present survey aimed to investigate the awareness of Italian parents of their child’s malocclusion and its correlation with their sociodemographic characteristics and background. Furthermore, the present survey aimed at assessing parental attitude towards orthodontic consultation and interventions.

## 2. Materials and Methods

### 2.1. Study Design

The survey was performed using a web-based questionnaire, consisting of 18-items, specifically developed by two specialists in orthodontics (R.B. and S.M.).

The included questions were similar to those of the questionnaire proposed by Hassan et al. [23], and adjustments were made according to the purpose of the present study.

The survey was pilot tested on a panel consisting of ten parents of school-aged children attending the Dental Unit of the AOU “San Giovanni di Dio e Ruggi D’Aragona” in Salerno (Italy), and the gathered feedback was used to make adaptations to the questions’ content and validity.

The language utilized throughout the questionnaire was intentionally kept simple and colloquial, ensuring accessibility to respondents of varied backgrounds, and facilitating clear and concise responses.

### 2.2. The Questionnaire: Structure and Content

The 18 questions of the questionnaire were divided into three sections (Supplementary Material File S1).

The first section (Q1–Q6) of the survey gathered the demographic information and characteristics of the respondents, such as relationship with the child (i.e., mother, father or other), level of education (i.e., primary school, high school or university), and marital status (i.e., single or partnered), and investigated whether parents had an history of orthodontic treatment, either themselves or their other sons or daughters (if any).

The second section (Q7–Q13) assessed the perception of the parents of their child’s orthodontic needs, namely their belief of a possible psychosocial impact of malocclusion and their attention to any child-focused dental (i.e., spaces between teeth, crowded anterior teeth, protruding upper teeth, extra or missing teeth, and incorrect teeth position), skeletal (i.e., facial asymmetry, protruding mandible, protruding upper jaw, and narrow palate) or functional problem (i.e., difficulty with speech, swallowing or chewing).

The third section (Q14–Q18) included questions about the parents’ attitude toward orthodontic consultation and treatment. Participants were asked whether they had already consulted a dentist and/or other specialists for their children, and if so, at what age and whether they had subsequently started a treatment.

### 2.3. Survey Dissemination and Terms of Participation

The survey was disseminated by a standardized recruitment electronic message made with Google Forms (https://www.google.com/forms/about/, accessed on 7 November 2022), including an Internet link to access the questionnaire and a cover letter, explaining the objective of the study and how the methods of data processing, ensuring respondents the anonymity of responses and requesting participation.

A complete list of all public primary schools in Campania (Italy) was obtained from the Campania Region website (https://www.regione.campania.it/, accessed on 14 November 2022). Schools were divided according to the province in which they were located (Naples, Benevento, Avellino, Caserta and Salerno), and three schools per province were randomly drawn through Randomizer.org, for a total of 15 schools.

The headmasters of these schools were contacted, requesting collaboration in spreading the questionnaire after explaining the purpose of the survey. Specifically, they were asked to post the standardized recruitment electronic message on the institutional website of the school they run, and to notify all parents of the post through the parental delegates of each class.

In case a school declined or did not respond, another school from the same province was randomly selected.

The terms of survey participation are listed below:

- participant must be 18 years or older;
- participant must be parent of a child between 6 and 11 years of age;
- participation was entirely voluntary, and each respondent could decide to stop filling out the questionnaire at any time, for any reason, without penalty;
- to ensure the anonymity of the responses, each participant was asked not to include his or her name or other information that could lead back to his or her identity in the responses;
- before answering the questions, each respondent was asked to consent to the processing of data in aggregate in accordance with EU Regulation n. 2016/679;
- no financial incentive was provided for participation in the survey;
- participants could only respond to the survey once, and it was not possible to modify responses after successfully submission;
- if participants had any doubts or questions about the nature of the research or about the questionnaire, they should contact the study investigators, whose name, surname, affiliation and email were given.

The schools’ headmasters were contacted on Monday, 19 December 2022.

Posts with recruitment messages were all published between 25 January 2023 and 10 February 2023.

The first reply to the questionnaire was recorded on Wednesday, 25 January 2023, at 09:27 am and response collection was terminated on Wednesday, 19 April 2023, at 09:31 pm.

### 2.4. Sample Size

The target sample size was estimated by the R Software, version 4.1.0 (May 2021, R Foundation for Statistical Computing, Vienna, Austria), using Cochran’s sample size formula for prevalence studies [24].

Since the total number of children in the age group between 6 and 11 in Italy is about 3 million, according to IStat data extracted on 27 November 2022, the target sample size for the survey was estimated to be 1067 participants, at a 95% confidence level and a 3% margin of error, considering two parents per child.

### 2.5. Statistical Analysis

Data obtained from the questionnaires were qualitatively summarized by a descriptive statistical analysis using Microsoft Excel software 2019 (Microsoft Corporation, Redmond, WA, USA), and frequencies and percentages were computed for each item.

Chi-square test was performed to assess association between responses. The associations investigated were the following: associations between participants’ characteristics and perceived child’s orthodontic needs; and associations between perceived child’s orthodontic needs and parents’ attitudes toward early orthodontic evaluation and interventions.

Statistical significance was set at *p*-value < 0.05. All statistical analyses were performed using SPSS software version 28.0 (IBM SPSS, Armonk, NY, USA).

## 3. Results

A total of 1806 parents participated in this survey, and their responses are summarized in Table 1.

The association between parental characteristics and perceived child’s orthodontic needs are shown in Table 2.

The awareness of the impact of children’s teeth on their personality was more widely shared among mothers than fathers (*p* < 0.001), among partnered parents than single ones (*p* < 0.001), and among parents who had a history of orthodontic treatment themselves or for another child than parents who did not (*p* < 0.05).

The thought that their child has a problem with the alignment/positioning of his/her teeth was more common in parents of older age than younger ones (<0.001), in parents with a higher level of education than less educated ones (*p* < 0.001), in partnered parents than single ones (*p* < 0.001), and in parents who underwent orthodontic treatment than ones who did not (*p* < 0.001).

The observation of child’s skeletal issues was more frequent in mothers than fathers (*p* < 0.001), in older parents than younger ones (*p* = 0.002), in partnered parents than single ones (*p* = 0.016), and in parents who underwent orthodontic treatment than ones who did not (*p* < 0.001).

Lastly, partnered parents more commonly observed functional problems than single ones (*p* = 0.005).

When analyzing the associations between perceived orthodontic needs and attitude toward orthodontic early interception, a positive association was found between the decision to consult a dentist or other specialists and the thought that their child has dental, skeletal or functional issues (Table 3). Similarly, the awareness of a potential psychological impact of teeth positively affected a parent’s decision to refer their children to dental or other specialists for examination, and to start an orthodontic treatment (Table 3).

Lastly, parents who first referred their children to an orthodontic evaluation after the age of 7 were more likely to see dental problems in their children (Table 3).

## 4. Discussion

Interceptive orthodontics groups a set of interventions for timely addressing orthodontic problems [25], and among these, parental education regarding the importance of time-appropriate orthodontic evaluation and interventions should be considered [22].

The present study aimed to investigate factors that potentially affect the awareness of a child’s orthodontic needs, influencing the attitude towards orthodontic evaluation and interventions among a sample of parents of school-aged children from Italy.

According to the results of the present survey, most parents were aware of the potential impact of teeth on their child’s personality, and this was consistent with the findings of previous studies [18,19,26]. This is in agreement with previous findings, supporting that dentofacial appearance has been recognized as a critically important factor in social interactions, which in turn affect personality [27,28]. This also applies to children, since it was found that those with a pleasant dental appearance are generally judged to be nicer looking, more desirable as friends, smarter and less likely to act aggressively [29].

Mothers seemed to be more concerned than fathers about the psychological impact of malocclusions, in agreement with the general tendency of mothers to be more focused on the emotional aspects of their children [30].

Two-thirds of the parents reported that their children had problems with the alignment of their teeth; this finding was in accordance with those of previous studies [18,21,31]. Crowded anterior teeth and protruding upper teeth were the most observed issues related to teeth alignment by these survey participants. Interestingly, these features were found to have a major negative impact on facial attractiveness [32,33]. Instead, only a minority of parents found functional problems in their children. Together, these observations suggest that parents’ perception of orthodontic needs is strongly linked to their attention to their child’s beauty. Accordingly, the number of parents seeking orthodontic treatment for their children has greatly increased in the last decade [34], as well as the attention to child’s beauty, which has increased because of the dissemination of messages that emphasize the importance of child’s physical appearance and portray children’s body stereotypes on social media [35].

Parents’ higher education and older age were associated with a greater awareness of orthodontic needs, in accordance with a previous finding from Assery et al. [36].

Moreover, a parental history of orthodontic treatment was found to be a crucial factor affecting both perception of children’s dental problems and the decision to consult an orthodontist. The reason why can be identified in the information related to possible dentoskeletal problems and intervention benefits that one acquires from attending an orthodontic specialist, enabling the development of greater attention to the child’s specific issues and greater trust in specialists [37,38].

Of the parents who took part of this study, about 60% had already referred their children to an orthodontic evaluation. Parents who perceived dental, skeletal, or functional issues were more likely to opt for an orthodontic evaluation. However, the small number of participants perceiving skeletal issues, notably narrow palate and protruding upper jaw, that the literature reported as most common orthodontic problems of pediatric age [7], pointed out the need for a better parental education regarding the importance of a specialistic evaluation. Indeed, a specialist can easily intercept any type of malocclusion, and not only the most visible to the layman’s eye [39].

Less than one-third of participants consulted other healthcare specialist for the dentofacial issues of their children. However, the attitude of consulting other specialists was significantly related to the parental thought that their children had functional problems. This finding suggests that parents are usually aware that some functional alterations associated with malocclusion, such as lingual interposition between arches, non-nutritive sucking habits, oral breathing and obstructive sleep apnea (OSA), are problems of speech, pediatric, and ENT interest, requiring multidisciplinary management [40,41].

Among parents who consulted an orthodontist, 44% referred their child to an examination after the age of 7. Otherwise, as previously mentioned, the AAO recommends that children have their first orthodontic evaluation before the age of 7 [42]. After this age, malocclusions are more likely to be moderate or severe, requiring longer and more complex therapies [7]. This is also the possible reason why parents who referred their children to an orthodontic examination after the age of 7 years old were found to have noted orthodontic problems more than those who consulted specialists before the age of 7 years. Since malocclusions are of a greater severity at this age, they are more likely to be perceived also the laypeople.

Moreover, the parents’ decision of initiating orthodontic treatment for their children was only affected by the belief of a significant impact of the teeth on their child’s personality, which is widespread among the participants who started the required therapy. However, other factors that have been recognized as potentially influencing parental decisions, such as economic conditions, health insurance, occupational background and the relation to their profession [43] were not investigated in the present study.

Considering the results of this study, some recommendations for improving parental awareness could be as follows:

- Raising awareness among pediatricians of the need for early orthodontic screening by a specialist;
- Organizing orthodontic screening and education campaigns in schools;
- Disseminating information messages with content validated by national and/or international scientific societies using the main communication media, including social media.

The results of the present study are generalizable within the following limitations.

First, a potential selection bias of participant should be considered since this study’s data collection was performed though a survey, whose participation was voluntary, and thus parents who were mildly concerned about the issue might have neglected it. Nevertheless, it may be conceivable that the self-administration of questions enables responses that truly reflect the beliefs of respondents. Second, the study included different parents belonging to the same Italian region, although from different social and cultural backgrounds. Lastly, some confounders were not considered, such as the socioeconomic characteristics of respondents, health insurance, and occupational background. However, the study also presented some strengths, since it augments the knowledge about parental awareness of their children orthodontic needs and helps to recognize characteristics of parents that are related to their attitude towards interceptive orthodontics.

Further investigations with larger samples are needed to confirm the present results. Moreover, since social media proved to have an increasing influence on subjects’ opinion formation [44,45], including health- and dental-care content [46,47], future studies should analyze the reliability and accuracy of information featured on sharing platforms regarding orthodontic needs and orthodontic treatment time, as well as their suitability for parental education.

## 5. Conclusions

The results of the current cross-sectional study confirmed the crucial role of parental educational level and a parental history of orthodontic treatment in parents’ awareness of orthodontic needs. Moreover, perceived orthodontic needs were found to be decisive in the attitude of seeking orthodontic consultation and beginning orthodontic treatment.

However, the present survey responses revealed the need for a better education of parents regarding the right time to initiate orthodontic periodic controls, which should become routine even in the absence of perceived problems.

## Figures and Tables

**Table 1:** Responses of participants to the questions of the present survey.

Question (Number of Respondents)	Answer Options	Response Count	Response Percentage
*Section 1. Demographic information and characteristics of the sample*			
Q1: Relation to the child (n = 1806)			
	Mother	1584	87.7%
	Father	222	12.3%
Q2: Age (n = 1806)			
	<30	144	8%
	30–40	858	47.5%
	>40	804	44.5%
Q3: Level of education (n = 1806)			
	Primary school	246	13.6%
	High school	792	43.9%
	University	768	42.5%
Q4: Marital status (n = 1806)			
	Single	432	24%
	Partnered	1374	76%
Q5: Personal history of orthodontic treatment (n = 1806)			
	Yes	1044	57.8%
	No	762	42.2%
Q6: Other children who underwent orthodontic treatment (n = 1806)			
	Yes	390	21.6%
	No	1416	78.4%
*Section 2. Perceived child’s orthodontic needs*			
Q7: Do you think that your child’s teeth would ever have a significant impact on his/her personality? (n = 1806)	Yes	1140	63.1%
	No	666	36.9%
Q8: Do you think your child has any problems with the alignment/positioning of his/her teeth? (n = 1806)	Yes	1122	62.1%
	No	684	37.9%
Q9: If yes, what problem is it? Choose one or more answers (n = 1122)	Spaces between teeth	432	38.5%
	Crowded anterior teeth	316	28.2%
	Protruding upper teeth	267	23.8%
	Extra teeth	32	2.9%
	Missing teeth	110	9.8%
	Incorrect teeth position	75	6.7%
Q10. Do you think your child has any skeletal problems? (n = 1806)	Yes	354	19.6%
	No	1452	80.4%
Q11: If yes, what problem is it? Choose one or more answers (n = 354)	Facial asymmetry	66	18.6%
	Protruding mandible	105	29.7%
	Protruding upper jaw	138	38.9%
	Narrow palate	45	12.7%
Q12: Do you think your child has any problems with oral functions? (n = 1806)	Yes	252	14.0%
	No	1554	14.0%
Q13: If yes, what problem is it? Choose one or more answers (n = 252)	Difficulty with speech	102	40.5%
	Difficulty in swallowing	72	28.6%
	Difficulty in chewing	114	45.2%
*Section 3. Attitude towards early orthodontic interventions*			
Q14: Have you ever referred your child to an orthodontic evaluation? (n = 1806)	Yes	1146	63.5%
	No	660	36.5%
Q15: Have you ever consulted other specialists for any of the previously listed problems? (n = 1806)	Yes	516	28.6%
	No	1290	71.4%
Q16: If yes, which one? Choose one or more answers (n = 516)	Pediatrician	414	80.2%
	Speech therapist	180	34.9%
	ENT	138	26.7%
	Osteopath	12	2.3%
Q17: If you answered yes to Q14, at what age did your child have the first orthodontic evaluation? (n = 1146)	Before the age of 7 years	508	44.3%
	After the of 7 years	638	55.7%
Q18: If you answered yes to Q14, after the consultation, did the child start an orthodontic treatment? (n = 1146)	Yes	755	65.9%
	No	391	34.1%

**Table 2:** Responses of the participants divided according to their characteristics regarding the perceived orthodontic needs for their children (n = 1806).

Question	Response	Q7: Do You Think That Your Child’s Teeth Would Ever Have a Significant Impact on His/Her Personality?		
		Yes	No	*p*-Value
Q1: Relation to the child	Mother	1062	522	<0.001 *
	Father	78	144	
Q2: Age	<30	96	48	0.100
	30–40	558	300	
	>40	486	318	
Q3: Level of education	Primary school	138	108	0.036
	High school	516	276	
	University	486	282	
Q4: Marital status	Single	330	102	<0.001 *
	Partnered	1038	336	
Q5. Personal history of orthodontic treatment	Yes	636	408	0.023 *
	No	504	258	
Q6. Other children who underwent orthodontic treatment	Yes	228	162	0.031 *
	No	912	504	
		**Q8: Do you think your child has any problems with the alignment/positioning of his/her teeth?**		
		**Yes**	**No**	***p*** **-value**
Q1: Relation to the child	Mother	984	600	0.991
	Father	138	84	
Q2: Age	<30	84	60	<0.001 *
	30–40	498	360	
	>40	540	264	
Q3: Level of education	Primary school	150	96	<0.001 *
	High school	450	342	
	University	522	246	
Q4: Marital status	Single	210	222	<0.001 *
	Partnered	912	462	
Q5. Personal history of orthodontic treatment	Yes	714	330	<0.001 *
	No	408	354	
Q6. Other children who underwent orthodontic treatment	Yes	252	138	0.252
	No	870	546	
		**Q10: Do you think your child has any skeletal problems?**		
		**Yes**	**No**	***p*** **-value**
Q1: Relation to the child	Mother	276	1308	<0.001 *
	Father	78	144	
Q2: Age	<30	12	132	0.002 *
	30–40	174	684	
	>40	168	636	
Q3: Level of education	Primary school	54	192	0.115
	High school	138	654	
	University	162	606	
Q4: Marital status	Single	102	330	0.016 *
	Partnered	252	1122	
Q5. Personal history of orthodontic treatment	Yes	234	810	<0.001 *
	No	120	642	
Q6. Other children who underwent orthodontic treatment	Yes	78	312	0.823
	No	276	1140	
		**Q12: Do you think your child has any problems with oral functions?**		
		**Yes**	**No**	***p*** **-value**
Q1: Relation to the child	Mother	216	1368	0.299
	Father	36	186	
Q2: Age	<30	12	132	0.110
	30–40	120	738	
	41–50	120	684	
Q3: Level of education	Primary school	36	210	0.129
	High school	96	696	
	University	120	648	
Q4: Marital status	Single	78	354	0.005 *
	Partnered	174	1200	
Q5. Personal history of orthodontic treatment	Yes	132	912	0.060
	No	120	642	
Q6. Other children who underwent orthodontic treatment	Yes	60	330	0.357
	No	192	1224	

* *p* < 0.05, significant association. Between-group differences were measured with the Chi-square test.

**Table 3:** Associations between perceived orthodontic needs and attitude towards early orthodontic evaluation and interventions, according to responses to Sections 2 and 3 of the questionnaire.

Question	Response	Q14: Have You Ever Referred Your Child to an Orthodontic Evaluation?		
		Yes	No	*p*-Value
Q7: Do you think that your child’s teeth would ever have a significant impact on his/her personality?	Yes	798	342	<0.001 *
	No	348	318	
Q8: Do you think your child has any problems with the alignment/positioning of his/her teeth?	Yes	942	180	<0.001 *
	No	204	480	
Q10: Do you think your child has any skeletal problems?	Yes	312	42	<0.001 *
	No	834	618	
Q12: Do you think your child has any problems with oral functions?	Yes	180	72	0.005 *
	No	966	588	
		**Q15: Have you ever consulted other specialists for any of the previously listed problems?**		
		**Yes**	**No**	***p*** **-value**
Q7: Do you think that your child’s teeth would ever have a significant impact on his/her personality?	Yes	390	750	<0.001 *
	No	126	540	
Q8: Do you think your child has any problems with the alignment/positioning of his/her teeth?	Yes	408	714	<0.001 *
	No	108	576	
Q10: Do you think your child has any skeletal problems?	Yes	204	150	<0.001 *
	No	312	1140	
Q12: Do you think your child has any problems with oral functions?	Yes	204	48	<0.001 *
	No	312	1242	
		**Q17: If you answered yes to Q14, at what age did your child have the first orthodontic evaluation?**		
		**<7 years old**	**>7 years old**	***p*** **-value**
Q7: Do you think that your child’s teeth would ever have a significant impact on his/her personality?	Yes	358	440	0.582
	No	150	198	
Q8: Do you think your child has any problems with the alignment/positioning of his/her teeth?	Yes	334	608	<0.001 *
	No	174	30	
Q10: Do you think your child has any skeletal problems?	Yes	136	176	0.758
	No	372	462	
Q12: Do you think your child has any problems with oral functions?	Yes	90	90	0.095
	No	418	548	
		**Q18: If you answered yes to Q14, after the consultation, did the child start an orthodontic treatment?**		
		**Yes**	**No**	***p*** **-value**
Q7: Do you think that your child’s teeth would ever have a significant impact on his/her personality?	Yes	551	247	<0.001 *
	No	204	144	
Q8: Do you think your child has any problems with the alignment/positioning of his/her teeth?	Yes	617	325	0.577
	No	138	66	
Q10: Do you think your child has any skeletal problems?	Yes	192	120	0.058
	No	563	271	
Q12: Do you think your child has any problems with oral functions?	Yes	120	60	0.809
	No	635	331	

* *p* < 0.05, significant association. Between-group differences were measured with the Chi-square test.

## Data Availability

The datasets used and/or analyzed during the current study are available from the corresponding author upon reasonable request.

## Supplementary Materials

The following supporting information can be downloaded at: https://www.mdpi.com/article/10.3390/clinpract14030092/s1, File S1: Perceived Orthodontic Needs and Attitude Towards Early Evaluation and Interventions—Questionnaire.
