# Supplementary material for: Perceived Orthodontic Needs and Attitudes towards Early Evaluation and Interventions: A Survey-Based Study among Parents of Italian School-Aged Children
Source: Clin Pract. 2024 Jun 18;14(3):1159–70. doi: 10.3390/clinpract14030092 (PMC11202826; doi:10.3390/clinpract14030092)
Supplement: Supplementary file 1 [file clinpract-14-00092-s001.zip › clinpract-3038195-supplementary.pdf]

Translated version.

SURVEY ON AWARENESS OF EARLY ORTHODONTIC INTERVENTION DIRECTED TO PARENTS OF SCHOOL-AGED CHILDREN

Dear Sir/Madam,

We ask you for a few minutes of your time to answer the questions in this survey, which is part of a study conducted by the University of Salerno.

Please remember that there are no right or wrong answers, but we invite you to answer on the basis of your knowledge and experience.

If you have more than one child, when answering the following questions please refer only to the child who attends primary school.

Thank you in advance for your cooperation.

* Indicates a required question

Privacy Policy

Please remember that your participation in the survey is voluntary and you may decide to stop filling out the questionnaire at any time, for any reason, without penalty. All survey responses will be kept anonymous and will not be traceable to your identity.

Therefore, please do not include your name or other information in your responses that could be traced back to your identity. The results of the study will be used for research purposes and not for clinical purposes. The data collected will be processed in aggregate in accordance with EU Regulation No. 2016/679*

The compilation does not involve any specific risk. If you feel fatigued, you can stop filling out and continue at another time.

If you have any questions or concerns about the nature of the research or about the survey, please contact the research managers.

Clicking on the "I Agree" button you acknowledge that:

- You have read and understand the information provided to you;
- You voluntarily agree to be a participant in this study and know that you can refuse to answer questions and/or withdraw from the research at any time, without having to specify your reasons;
- You are at least 18 years old.
- You have at least one child between the ages of 6 and 11.

○ I agree

Section 1 – Sociodemographic characteristics of the respondents

1. What is your relationship with the child? *

- O Mother
- O Father
- O Child's guardian

2. How old are you? *

- O < 30
- O 30-40
- O 41-50
- O > 50

3. What is your highest level of education? *

- O Primary school
- O Secondary school (middle school)
- O High school diploma
- O Enrolled in university, but not graduated
- O Degree
- O Master/PhD

4. What is your marital status? *

- O Married/Engaged
- O Divorced/Separated

5. Have you ever undergone orthodontic treatment yourself?*

- O Yes
- O No

6. If you have other children, have they undergone orthodontic treatment? *

- O Yes
- O No

Section 2 – Perception of problems and orthodontic needs

7. Do you think that your child's teeth would ever have a significant impact on his/her personality?*

- O Yes
- O No

8. Do you think your child has any problems with the alignment/positioning of his/her teeth?*

- ○ Yes
- ○ No

9. If yes, what kind of problem did you notice? (Choose one or more answers)

- ○ Spaces between teeth
- ○ Crowded teeth (lack of space)
- ○ One or more missing teeth
- ○ Presence of extra teeth
- ○ Protruding upper teeth ("buck teeth" or "bunny teeth)
- ○ Space between upper and lower teeth
- ○ Other: ______________________

10. Do you think your child has any skeletal problems? *

- ○ Yes
- ○ No

11. If yes, what kind of problem did you notice? (Choose one or more answers)

- ○ Facial asymmetry (chin deviating to the right or left)
- ○ Protruding chin
- ○ Protruding upper jaw
- ○ Other: ________________

12. Do you think your child has any problems with oral functions? *

- ○ Yes
- ○ No

13. If yes, what kind of problem did you notice? (Choose one or more answers)

- ○ Speech difficulties
- ○ Swallowing difficulties
- ○ Chewing difficulties
- ○ Other: ________________

Section 3 – Attitude toward orthodontic consultation and treatment

14. Have you ever take your child to an orthodontic evaluation for any of the previously listed problems?*

- ○ Yes
- ○ No

15. Have you ever consulted other specialists for any of the previously listed problems?*

- ○ Yes
- ○ No

16. If yes, which one? (Choose one or more answers)

- ○ Pediatrician
- ○ ENT specialist
- ○ Speech therapist
- ○ Osteopath
- ○ Posturologist
- ○ Physiotherapist
- ○ Other: _______________

17. If you answered yes to Q14, at what age did your child have the first orthodontic evaluation? *

- ○ By the age of 7 years
- ○ After the age of 7 years

18. If you answered yes to Q14, after the consultation, did your child start an orthodontic treatment? *

- ○ Yes
- ○ No
